# Lung and chest wall volume during vital capacity manoeuvre in Osteogenesis Imperfecta

**DOI:** 10.1186/s13023-022-02535-y

**Published:** 2022-10-28

**Authors:** Antonella LoMauro, Davide Lacca, Vittorio Landoni, Andrea Aliverti

**Affiliations:** 1 Dipartimento di Elettronica, Informazione e Bioingegneria, Politecnico di Milano, piazza Leonardo Da Vinci, 20133 Milan, Italy; 2 Valduce Hospital - Villa Beretta Rehabilitation Centre, Lecco, Italy

**Keywords:** Osteogenesis Imperfecta, Spirometry, Opto-electronic plethysmography, Vital capacity, Thoraco-abdominal volume

## Abstract

**Background:**

Although Osteogenesis Imperfecta (OI) affects the connective tissue, pulmonary function might be compromised because of thoracic deformities. OI is known to be a restrictive lung disease, but spirometry provides global measurement without localizing the site of the restriction. Opto-electronic plethysmography (OEP), is a non-invasive method able to underline altered respiratory function as well as ventilatory thoraco-abdominal paradoxes during spontaneous breathing. We aimed to reconstruct the thoraco-abdominal surface, to perform local analyses of trunk motion and to make quantitative comparison of trunk shape and respiratory kinematics according to OI severity, particularly during maximal inspiratory and expiratory expansions. This is a cross-sectional study where we have studied the thoraco-abdominal compartmental analysis in 26 adult OI patients (14 Type III) at rest and during vital capacity manoeuvre using OEP. We have also applied a new method that created realistic and accurate 3D models to perform local analyses of trunk motion and to make quantitative comparison of trunk shape and respiratory kinematics.

**Results:**

Type III patients were characterized by lower spirometric lung volume, by lower sleep quality, by a more compressed thoracic configuration aggravated by severe scoliosis, by reduced global expansion at rest and during maximal maneuvers because of the reduced expansion of the pulmonary ribcage at rest (12% vs. 65% in healthy subjects), during maximal inspiration (37% vs. 69%) and expiration (16% vs. 68%) with local paradoxical movement occurring on the side of the ribcage region.

**Conclusion:**

The kinematics of the trunk changed to compensate for the severe structural deformities by shifting the expansion in the abdomen both at rest and during maximal manoeuvre because of a restricted thorax. For the first time, we have quantified and localized the site of the restriction in OI patients in the lateral part of the thorax. The 3D analysis proposed seemed a promising graphical immediate new method for pathophysiology study of chest wall restriction.

## Introduction

Although Osteogenesis Imperfecta (OI) is a rare genetic disorder affecting the connective tissue [1–3], in particular type I procollagen, respiration is one of the functions that are mostly affected. Because type I collagen is widely expressed in the human body and it is a component of the extracellular matrix of many tissues and organs, individuals with OI manifest also extra skeletal features including pulmonary disease [4].

In addition, the deformities at the rib cage level induced by OI usually reduce the patients’ ability to breathe properly. Because respiratory failure is the leading cause of death in OI [5–8], it is of paramount importance to study breathing function in these patients.

The majority of works in literature investigated the restriction effect of OI on lung function. Individuals with the more severe form of the disease, OI type III [9, 10], have significantly reduced forced vital capacity (FVC) and forced expiratory volume in one second (FEV_1_) which do not follow the expected trends of the normal population [11–16]. Although being one of the most important clinical investigation, spirometry might lead to misleading results in OI due to the disproportional growth that might affect the prediction equation resulting in underestimating the pulmonary involvement, particularly in severe OI patients [14, 15]. In addition, spirometry provides global measurement, therefore not allowing to localize the site of the restriction.

For this reason, it is important to consider and develop other methods to examine the breathing function of these patients with another prospective and at different levels of localization. However, there are few works that investigated the effect of chest wall geometry on the respiratory function. We have previously studied the influence of rib cage deformities on respiration in subjects with severe OI. Using opto-electronic plethysmography (OEP), the rib cage geometry was studied, together with the ventilatory pattern, in seated and supine position. The alteration of the respiratory function seemed to be correlated to the severity of the disease. The great rib cage and sternal deformities caused the inefficiency of respiratory muscles, potentially leading to breathing difficulties. Ventilatory paradoxes and asynchronies among chest wall compartments were also present [15]. However, we applied the OEP method only to spontaneous breathing. No information is therefore available on the thoraco-abdominal strategies adopted by these patients during maximal chest wall (and lung) expansion.

A morphological analysis on OI patients was recently conducted by Sanchis-Gimeno et al. in order to evaluate the influence of ribs shape on respiratory function. Starting from CT images at maximal inspiration and maximal expiration, the authors were able to build 3D virtual models of the rib cage and of the thoracic spine. A set of landmarks was superimposed to the models to extract information about the size of the bones. The shape of the ribs and the spine was correlated to the ventilatory manoeuvre performed by the subjects. The results showed that OI patients, who had more horizontally aligned ribs than healthy controls, presented lower values of FVC and FEV_1_ [17]. Although providing highly detailed images, especially of bone structures, CT is an invasive imaging method since it is based on ionizing radiations. We have just developed and validated a new quantitative analysis by 3D graphics of thoraco-abdominal surface shape and breathing motion based on OEP and therefore on non-ionizing radiations. The methods was proposed on healthy subjects and on two cases [18].

For this reason, the aims of this work were to extend the analysis of thoraco-abdominal volume to vital capacity manoeuvre and to apply to OI the new method that non-invasively created realistic and accurate 3D models of the trunk.

Starting from OEP data, based on non-ionizing radiations, we aimed to precisely reconstruct the thoraco-abdominal surface, to perform local analyses of trunk motion and to make quantitative comparison of trunk shape and respiratory kinematics according to OI severity.

## Study design and methods

This cross-sectional study was conducted according to the statement of the Declaration of Helsinki. The use of data for publication was approved by the Ethics Board of Politecnico di Milano, Italy (Parere n. 47/2021).

### Subjects

OI patients were enrolled among the members of As.It.O.I., the Italian Association of Osteogenesis Imperfecta, by posting an announcement on the association Facebook page. Confirmed diagnosis of OI type III and IV, stable condition and absence of severe cardio-respiratory pathologies were the inclusion criteria.

The healthy control group was recruited among colleagues and friends of the authors and it was composed to reflect the distribution of the OI group in terms of gender and age. Stable condition and absence of severe cardio-respiratory pathologies were the inclusion criteria.

#### Informed consent

was obtained from all study subjects or parents.

### Spinal and ribcage geometry

Patients underwent standard anteroposterior and lateral radiographic views of the entire spine and the thoracic and lumbar scoliosis were quantified using the Cobb method [19, 20].

The geometry of the 3D models was quantified in all subjects at end-expiration. The quantities that have been measured were: absolute volumes of the total chest wall and its three compartments (namely, pulmonary rib cage, abdominal rib cage and abdomen), expressed as absolute values and as percentage of the chest wall volume. Areas of the total chest wall and of the transversal sections (created by cutting the model with three planes at the reference heights) at angle of Louis, xiphoid and umbilical levels; trunk height, measured along the longitudinal axis between top and bottom central markers; Antero-Posterior (AP) and Medio-Lateral (ML) diameters at angle of Louis, xiphoid and umbilical levels, measured as distances between couples of specific markers; and perimeters of the sections at angle of Louis, xiphoid and umbilical levels were also computed. Starting from the previously mentioned parameters, a set of other variables was derived: the ratio between AP and ML diameters, computed at the three reference levels, the surface-to-volume ratio, and the ratio between trunk height and perimeter at the xiphoid level. All these variables were considered as a set of shape factors to characterize the trunk’s geometry.

### Lung function and nocturnal oxygen saturation

Measurement of FVC, FEV_1_, peak expiratory flow, peak cough flow and total lung capacity (TLC), assessed through the nitrogen washout technique, were performed in all patients (Vmax series 22, SensorMedics, Yorba Linda,CA). The predicted values were computed according to the Global Lung Initiative [21, 22].

Mean nocturnal oxygen saturation was measured using a digital pulse oximeter (Nonin, 8500 digital pulse oximeter Quitman, TX) and the number of apnoea and hypopnea events per hour of sleep (AHI index) as well as the number of times per hour of sleep of oxygen desaturation (ODI index) calculated. To be registered as an event, an apnoea or hypopnea must last at least 10 s or longer. AHI index is obtained by dividing the total number of apnoeic and hypopnea events by the total number of hours of sleep [23]. Similarly, ODI index is defined when blood oxygen levels fall below normal for 10 s or longer [24]. These parameters were obtained through at-home sleep apnoea test, that includes a small finger probe to measure oxygen levels and an oronasal thermal airflow sensor to estimate airflow and breathing.

### Optoelectronic plethysmography

Optoelectronic Plethysmography (OEP) is a non-invasive measurement technique that allows for 3D tracking of body movements [25]. OEP exploits a set of infrareds reflective markers, placed on the skin of subjects according to precise anatomical reference points. The thoraco-abdominal volumes can be calculated from markers’ positions through surface triangulation and successive integration. OEP accuracy has been established and improved over time [26].

All the subjects were in seated position during the acquisition process. Firstly, they were asked to breathe normally (i.e.: tidal volume, VT) and then to perform a maximal manoeuvre: a full inspiration (i.e.: inspiratory capacity, IC) followed by a full expiration (i.e.: vital capacity, VC). The number of markers placed on the subjects was variable according to the dimensions and the degree of deformity of the trunk. For most of the patients and for the control group, 89 markers were used. In smaller patients, a 63 markers configuration was used. Finally, a 52 markers configuration was used for two subjects who were not able to hold the seated position without the help of a chair. The configuration was the same of the 89 markers, except for the markers on the back that were absent since the presence of the chair did not allow their acquisition [27].

The volume variation of the chest wall as well of its three compartments were calculated as absolute and percentage values. The product of tidal volume and respiratory rate (RR) gives minute ventilation (MV), that represents the exchange of gas per minute.

### Models creation

The proposed method was previously described in detail [18]. In brief, starting from the OEP output file, the 3D markers’ coordinates were extracted to generate the coordinates matrix. The markers configurations were then combined with a triangulation matrix, defined by the same protocol used to place the markers on the subjects. A smoothing procedure was applied. It consisted in an automatic and iterative interpolation process that increased the number of vertices from 93 to 548. The level of detail obtained after the smoothing allowed for precise local analysis of the thoraco-abdominal surface. A series of measurements can be performed in order to characterize the geometry of the trunk and its three compartments. These comprised: volumes and volumes variations, trunk height and diameters, perimeter and area of transversal sections, angle at the sternum, minute ventilation. Three reference levels were defined along the longitudinal direction: angle of Louis, xiphoid, and umbilicus. Some shape factors were also computed, such as surface-to-volume ratio or height-to-perimeter ratio. Since all the models presented the same number of points and the correspondence between each of them was maintained, it was possible to build the vector field associated to the breathing motion of all the vertices, in terms of the magnitude and the direction of motion. The vector field (VF) data could be analysed and displayed through two graphic tools: a 3D heatmap, in which the magnitude of motion was associated to different colours, and a 3D arrow plot, that allowed to visualize both the magnitude and the direction of motion with color-coded arrows.

### Statistical analysis

Sample size calculation was based on previously published FVC mean and standard deviation values in OI type III and IV [15]: difference between these two independent means provided an effect size of 1.571 that, with a type-1 error probability α = 0.05, a power (1-β, with β being type-2 error probability) = 0.95 and an allocation ratio = 1, resulted in a sample size of 20 subjects (10 type III and 10 type I-IV, G*Power 3.1.9.4 software).

Comparative analyses were performed among the three groups, with OI patients grouped according to disease severity (III vs. I and IV). Since the number of subjects within each group was limited, the distributions of the measured parameters were not normal, according to the normality test. For this reason, median, quartiles, and interquartile range were computed for all the variables and the Kruskal-Wallis test of hypotheses was performed with group as independent variable. The significance level was set at 95%.

To better compare the average displacements, the colour maps were normalized between maximum and minimum VF values and the statistical analysis of the data was also performed. A vector of p-values was then obtained and used to represent the test results directly on the 3D model. Starting from an averaged and uncoloured 3D model obtained from all the subjects belonging to each group, only the points with the p-value below (i.e.: p-value < 0.05) the threshold were coloured. In particular, two colours were used: red for points where the first group presented a greater average displacement than the second group, and blue for the opposite situation [18]. Data in text are reported as median (interquartile range).

## Results

### Subjects

Overall, 26 OI patients were enrolled (19 females and 7 males), together with 16 controls (11 females and 5 males) of similar age (median age: 33.5 years; first quartile: 29.5 years; third quartile: 44.7 years, p = 0.919).

Considering the OI forms and severity, there were 14 patients affected by the most severe non-lethal form (Type III) and 12 by milder forms (6 Type I and 6 Type IV), similar in age and body mass index although the former were characterized by lower weight and height (Table 1). The controls were taller (median height: 1.69 m; first quartile: 1.59 m; third quartile: 1.77 cm, p < 0.001) and heavier (median weight: 65 kg; first quartile: 51 kg; third quartile: 78 kg, p < 0.01) than OI patients.

**Table 1:** Anthropometry, scoliosis, lung volume and nocturnal oxygen saturation

	OI Type III			OI Type I-IV			
	median	1st Q	3rd Q	median	1st Q	3rd Q	p-value
**Age (years)**	37.0	32.7	45.8	33.3	17.2	44.5	0.409
**Weight (kg)**	34.6	27.55	46.2	58.0	46.8	66.2	**< 0.001**
**Height (m)**	1.03	0.99	1.15	1.39	1.33	1.45	**< 0.001**
**BMI (kg/m** ^**2**^ **)**	30.3	25.4	34.0	30.9	24.8	36.9	0.564
**Thoracic scoliosis (Cobb angle)**	30.0	30.0	40.0	30.0	20.0	30.0	0.083
**Lumbar scoliosis (Cobb angle)**	30.0	30.0	40.0	20.0	20.0	30.0	**0.018**
**FVC (L)**	0.92	0.68	1.33	2.23	2.11	2.55	**<0.001**
**FVC (% predicted)**	66.4	46.1	79.6	82.3	74.6	90.8	**0.059**
**FEV**_**1**_ **(L)**	0.73	0.51	1.09	1.88	1.60	2.44	**<0.001**
**FEV**_**1**_ **(% predicted)**	66.0	40.9	80.9	79.6	69.2	95.0	**0.026**
**FEV**_**1**_ **/FVC (%)**	77.9	72.7	86.8	84.7	80.5	86.2	0.436
**TLC (L)**	1.43	1.12	2.12	3.05	2.55	3.28	**< 0.001**
**TLC (% predicted)**	69.3	59.0	85.6	78.6	72.9	90.1	0.236
**PEF (L/min)**	180	150	250	290	270	300	**0.002**
**PCF (L/min)**	250	190	370	445	375	465	**0.004**
**AHI (events/hour)**	10.0	4.0	17.4	2.3	1.0	6.7	**0.012**
**ODI (events/hour)**	10.3	4.2	18.6	3.3	1.1	7.0	**0.017**
**mean nocturnal SaO**_**2**_ **(%)**	93.2	91.0	94.2	95.2	93.5	95.6	**0.006**

AHI: apnea-hypoapnea index; BMI: body mass index; FEV_1_: forced expiratory volume in 1 s; FVC: forced vital capacity; ODI: oxygen desaturation index; PCF: peak cough flow; PEF: peak expiratory flow; TLC: total lung capacity.; 1st Q: first quartile; 3rd Q: third quartile

### Spinal and ribcage geometry

The sum of thoracic and lumbar scoliosis was significantly higher in Type III patients (median: 60°; 25th percentile: 60°; 75th percentile: 80°; p = 0.016) compared to Type I-IV patients (median: 40°; 25th percentile: 15°; 75th percentile: 60°), particularly because significant lumbar scoliosis (Table 1).

Table 2 reported the analysis of the geometry of the chest wall and its three compartments. Type III patients showed the lowest values of total and compartmental absolute volumes, as well as of trunk surface area, of trunk height, of perimeter and area at Louis Angle; and of trunk height normalized according to the perimeter at xiphoidal process. In these patients, the thoraco-abdominal distribution was different, with significantly higher abdominal percentage.

**Table 2:** Median (interquartile range) of chest wall geometry parameters

		Healthy	OI Type III	OI Type I-IV	Healthy vs. OI Type III	Healthy vs. OI Type I-IV	OI Type III vs. OI Type I-IV
**volume**	**pulmonary rib cage [L]**	10.23 (3.29)	4.41 (4.71)	9.2 (2.28)	**0.001**	0.227	**0.005**
	**pulmonary rib cage [% chest wall]**	51.5 (8.4)	48.8 (9.1)	55.1 (20.4)	0.198	1.000	0.382
	**abdominal rib cage [L]**	3.24 (2.1)	2 (1.25)	2.85 (2.33)	**0.006**	0.307	0.123
	**abdominal rib cage [% chest wall]**	18.8 (6.2)	15.7 (5.9)	15.4 (10)	0.561	0.378	0.328
	**abdomen [L]**	5.42 (3.06)	3.35 (3.62)	5.34 (5.16)	**0.046**	0.853	0.181
	**abdomen [% chest wall]**	29.1 (6.5)	34.1 (7.5)	28.3 (13)	**0.051**	0.853	0.572
	**chest wall [L]**	20.07 (7.24)	9.89 (9.92)	18.1 (11.66)	**0.005**	0.642	**0.018**
	**trunk height [cm]**	39.4 (4.3)	23.1 (4)	30.8 (5.3)	**0.000**	**0.000**	**0.000**
**diameters**	**AP at angle of Louis level [cm]**	18.5 (2.6)	18.2 (5.1)	19.2 (3.3)	0.868	0.246	0.304
	**AP at xiphoid level [cm]**	22.9 (3.2)	23.7 (6.9)	26 (4.8)	0.318	**0.041**	0.504
	**AP at umbilical level [cm]**	22 (4.5)	27.2 (8.3)	26.2 (13.1)	0.067	**0.023**	0.837
	**ML at angle of Louis level [cm]**	26.8 (3.4)	22.7 (8.3)	27.5 (4)	0.212	0.265	0.111
	**ML at xiphoid level [cm]**	27.2 (2.9)	25.5 (6.7)	29.5 (5.1)	0.868	0.137	0.217
	**ML at umbilical level [cm]**	26.7 (4.1)	26.4 (6.4)	29 (8.5)	0.835	0.246	0.355
	**AP/ML at angle of Louis level**	0.7 (0.11)	0.75 (0.19)	0.71 (0.05)	0.124	0.676	0.136
	**AP/ML at xiphoid level**	0.86 (0.1)	0.9 (0.1)	0.89 (0.05)	0.280	0.150	0.959
	**AP/ML at umbilical level**	0.83 (0.05)	0.99 (0.17)	0.9 (0.14)	**0.000**	**0.002**	0.504
**perimeter**	**at angle of Louis level [cm]**	87.3 (10.8)	72.3 (16.3)	85.8 (14.2)	**0.031**	0.676	0.080
	**at xiphoid level [cm]**	87 (10.6)	82.1 (24.1)	93.1 (28.8)	0.739	0.194	0.258
	**at umbilical level [cm]**	82.1 (14.5)	85.2 (24.5)	94.5 (34.2)	0.339	0.095	0.537
**area**	**chest wall [cm** ^**2**^ **]**	4168 (979)	2514 (1488)	3826 (1652)	**0.002**	0.378	**0.016**
	**at angle of Louis level [cm** ^**2**^ **]**	527 (149)	390 (182)	524 (159)	**0.038**	0.745	0.100
	**at xiphoid level [cm** ^**2**^ **]**	576 (145)	507 (354)	657 (407)	0.868	0.137	0.258
	**at umbilical level [cm** ^**2**^ **]**	508 (197)	560 (351)	678 (511)	0.406	0.078	0.440
**ratio**	**chest wall area/ chest wall volume [cm** ^**− 1**^ **]**	0.21 (0.03)	0.25 (0.06)	0.21 (0.04)	**0.009**	0.781	**0.014**
	**trunk height/perimeter at xiphoid level**	0.46 (0.05)	0.28 (0.06)	0.31 (0.1)	**0.000**	**0.000**	**0.057**

AP: Antero-Posterior; ML: Medio-Lateral

### Lung function and nocturnal oxygen saturation

All the measured lung volumes and flows were significantly lower in Type III patients who were also characterized by higher AHI and ODI resulting in reduced mean nocturnal saturation. The restriction of Type III patients was evident with volumes expressed both as absolute value and as percentage of the predicted values (Table 1). Type III patients were also characterized by mild obstructive sleep apnoea, as 5 ≤ AHI < 15 [28].

### Tidal volume

**Fig. 1:**
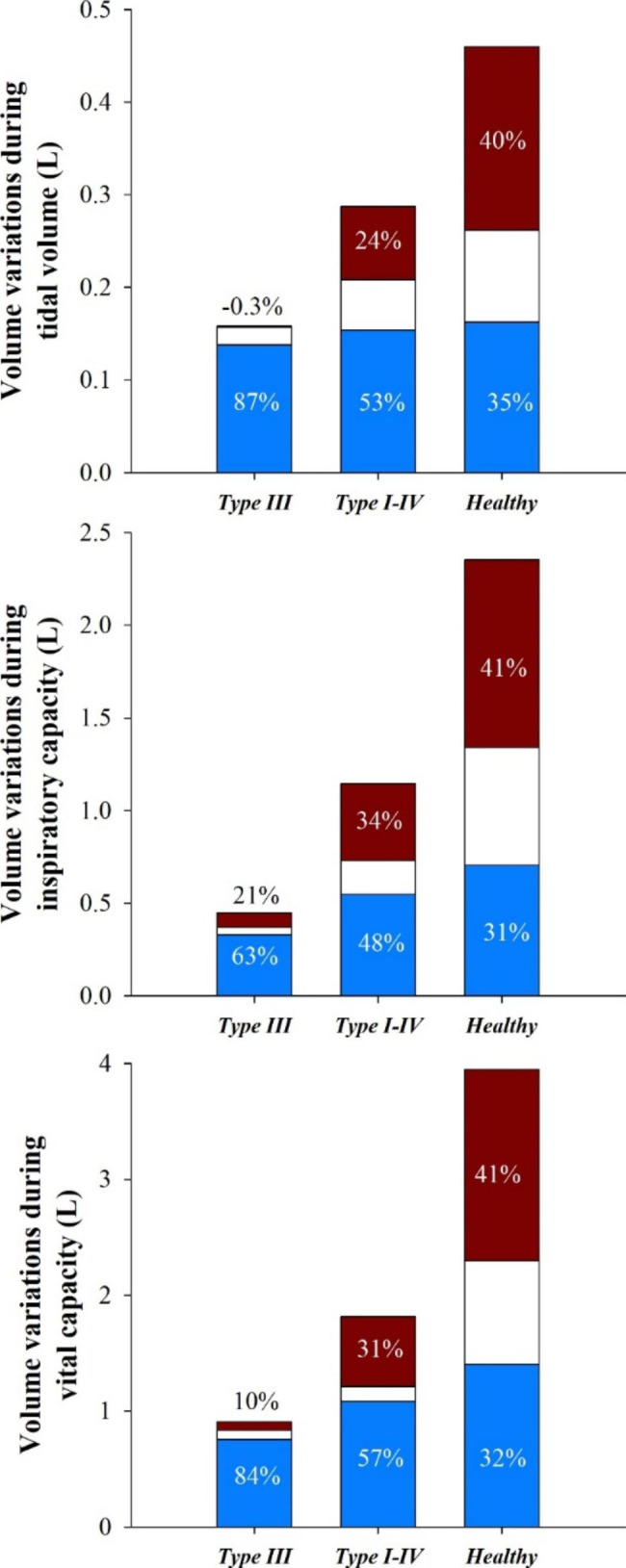
Stacked bar graph of pulmonary ribcage (red), abdominal ribcage (white) and abdominal (bleu) volume variation during tidal volume (top), inspiratory capacity (middle) and vital capacity (bottom) in Type III patients, Type I-IV patients and healthy subjects. The numbers represent the corresponding percentage contribution of each compartment to total chest wall volume variation

While the RR was similar (p = 0.109) in OI Type III, Type I-IV and controls (20.7 (11.6); 19.4 (5.5) and 17 (3.5) breaths/min, respectively), the tidal volume was halved (0.19 (0.08); 0.29 (0.19) and 0.47 (0.09) L; p < 0.001 controls vs. OI patients). As also shown in Fig. 1, Type III patients showed the lowest values of tidal volume also compared to Type I-IV patients (p = 0.001). This resulted in a halved Minute Ventilation (8.23 (2.1) L/min in healthy subjects vs. 3.72 (1.6) L/min in Type III OI patients vs. 5.1 (2.2) in Type I-IV OI patients; p < 0.001) and in OI patients breathing with a significantly higher rapid and shallow breathing index (112.9 (64.3) breaths/min·L in Type III and 76.9 (60.3) breaths/min·L in Type I-IV) compared to healthy subjects (35.5 (16.1) breaths/min·L; p < 0.001).

The expansion of the pulmonary ribcage was significantly different among the three groups (p < 0.001) when expressed as both absolute value and as percentage of tidal volume. The expansion of the abdominal ribcage was significantly different among the three groups (p < 0.001) when expressed as absolute value, but not as percentage of tidal volume. The expansion of the abdomen was similar among the three groups when expressed as absolute value, while it was significantly higher (p < 0.001) in Type III patients as percentage contribution to tidal volume (Fig. 1, top panel).

### Inspiratory capacity

Full inspiration was lower in patients (p < 0.001). The expansion of the two ribcage compartments was different among the three groups (p < 0.001) when expressed as absolute value; while it was significantly lower (p = 0.0088) only in Type III patients compared to healthy control when expressed as percentage of IC. The expansion of the abdomen was different (p < 0.05) among the three groups when expressed as absolute and percentage values. However, Type III patients were characterized by the lowest abdominal expansion, but the highest abdominal contribution to IC (Fig. 1, middle panel).

### Vital capacity

The total VC volume of the control group was in average more than twice the VC of OI patients (3.88 L vs. 1.26 L, respectively). In the case of Type III OI more than 80% of VC came from the abdomen (compared to 32% in controls), although with the lowest absolute expansion in all compartments (p < 0.001). Compared to healthy controls, Type I-IV patients were characterized by lower thoracic expansion (p < 0.001), but similar abdominal expansion (p = 0.104).

### Vector field analysis

**Fig. 2:**
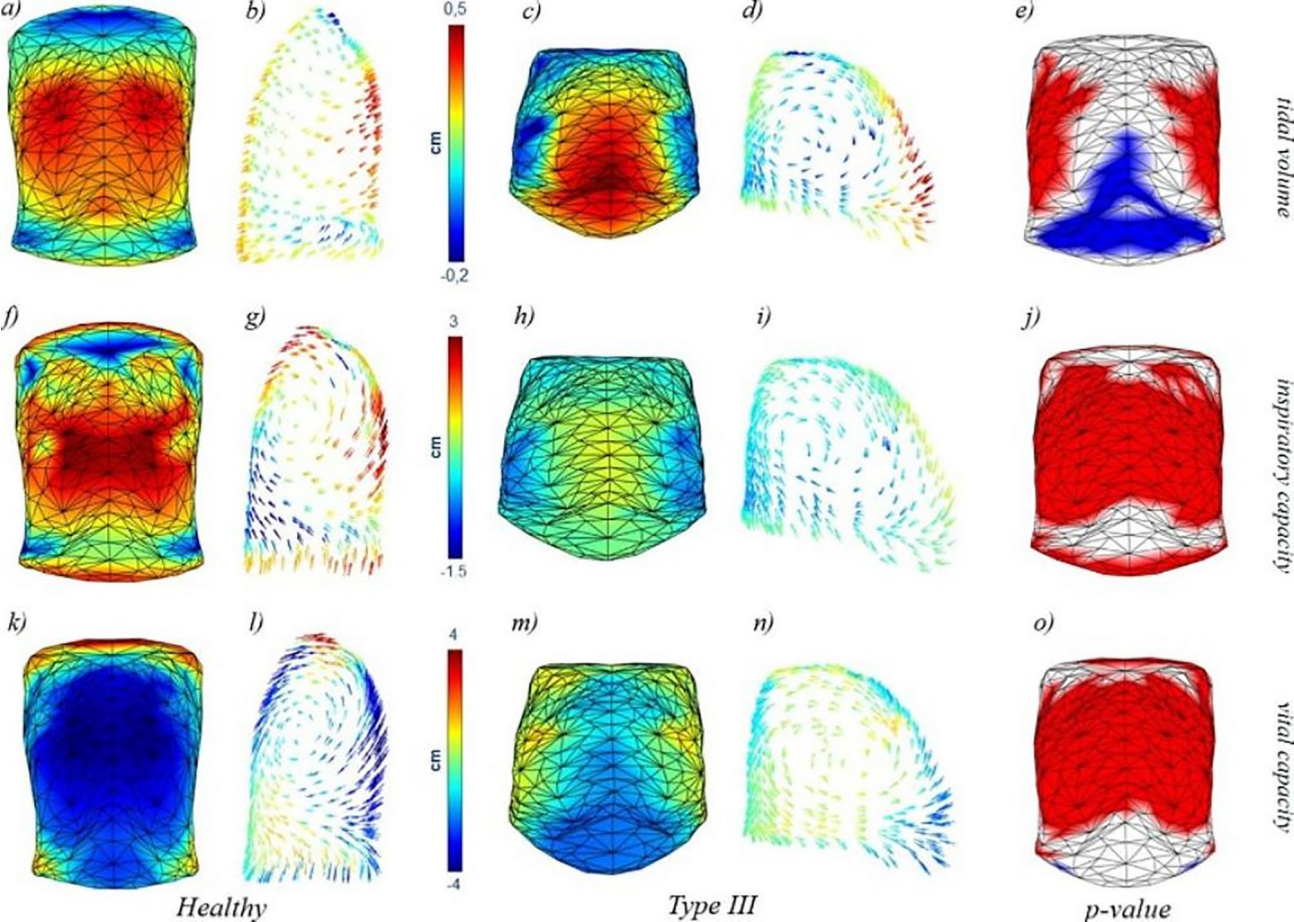
Colormaps (a, c, f, h, k, m) and arrow plots (b, d, g, i, l, n) of healthy subjects and OI Type III patients for the three volume variations considered: Tidal Volume (a-d), Inspiratory Capacity (f-i), and Vital Capacity (k-n). Statistical colormaps of the p-value are represented (e, j, o) with red colour indicating points where the healthy subjects presented a greater average displacement than OI Type III patients, and blue for the opposite situation

**Fig. 3:**
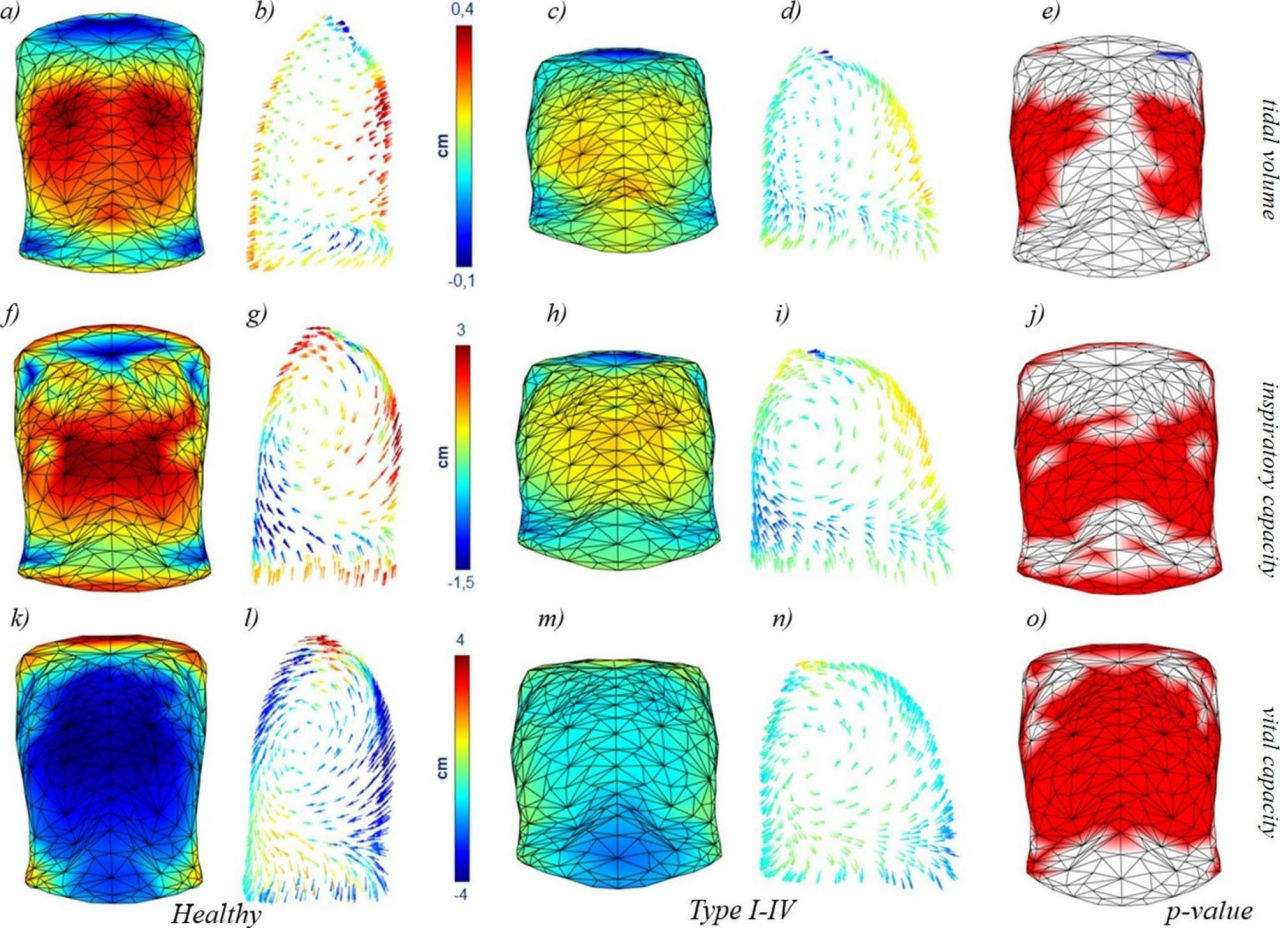
Colormaps (a, c, f, h, k, m) and arrow plots (b, d, g, i, l, n) of healthy subjects and OI Type I-IV patients for the three volume variations considered: Tidal Volume (a-d), Inspiratory Capacity (c, d), and Vital Capacity (e, f). Statistical colormaps of the p-value are represented (e, j, o) with red colour indicating points where the healthy subjects presented a greater average displacement than OI Type I-IV patients, and blue for the opposite situation

In general, OI patients presented a significant and lower displacement involving almost all the trunk (front and back) with higher contribution of the abdomen while the control group presented more prominent motion on the sides of the rib cage and on the back. More in detail, the colormap representing the average motion during VT showed the lateral inspiratory paradox in OI Type III patients (blue areas represent a negative or inward displacement, Fig. 2c). The paradox is slightly reduced during Inspiratory Capacity (Fig. 2 h). The average 3D models represented in Fig. 2 showed the different trunk shapes of controls and patients, with the OI Type III group being characterized by a barrel-shaped chest wall. The arrow plots of Fig. 2 showed that the chest wall motion in OI Type III patients assumed a direction that was more vertical and almost parallel to the thoraco-abdominal surface in the upper part of the rib cage, while it was similar to healthy subjects in the abdominal region. During VC, the yellow area indicated that lateral paradox was present also in the expiratory phase (Fig. 2 m). The motion was therefore directed towards the outside, enlarging the rib cage instead of reducing its volume. On the other hand, OI Type I-IV patients presented milder differences, with the magnitude of motion being lower than the control group during IC and VC. In the quiet breathing phase, the displacement of the sides and the back in healthy subjects were also larger, but this time there was no significant contribution of the abdomen for OI Type I-IV patients (Fig. 3).

**Fig. 4:**
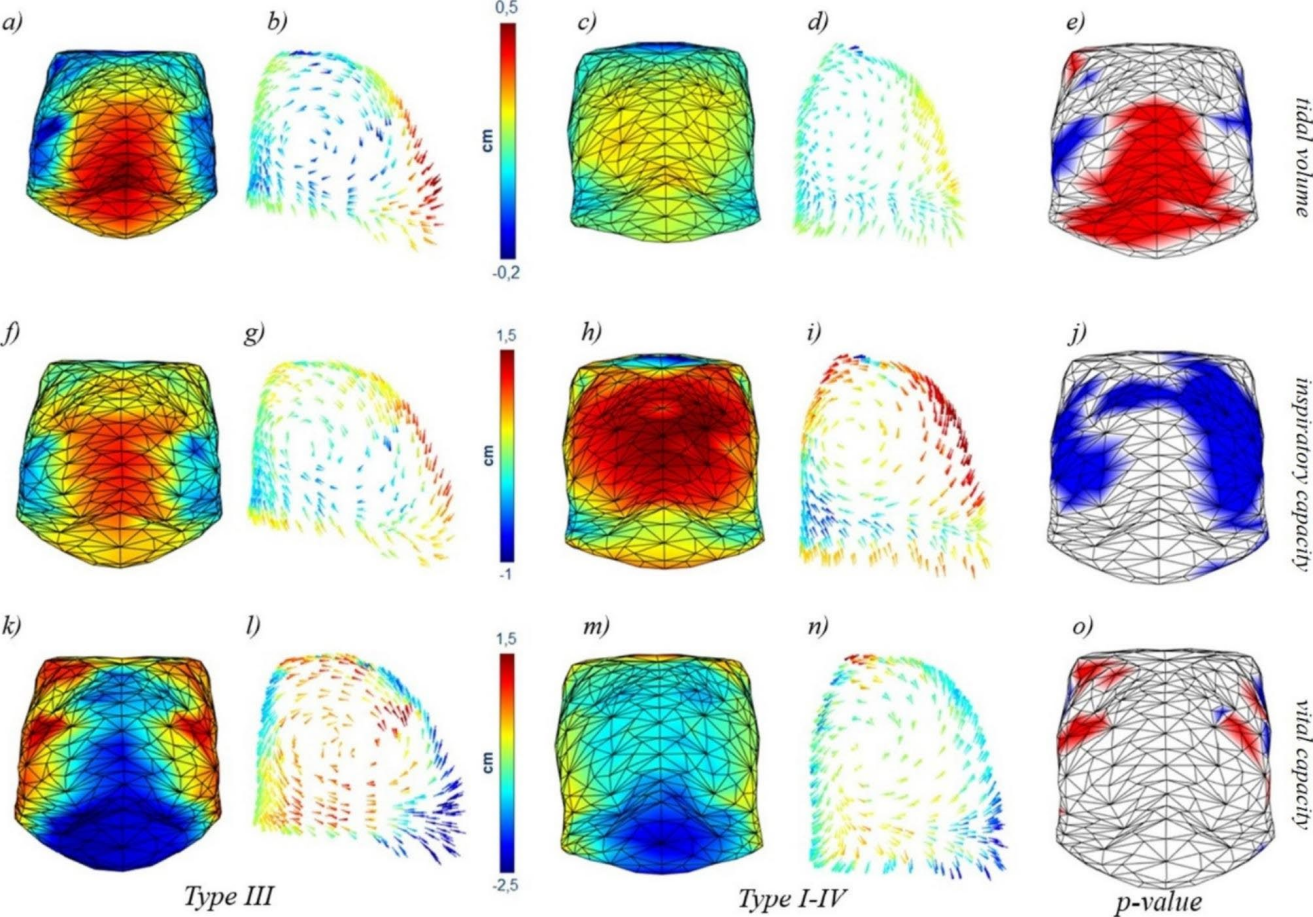
showed the difference the two groups of OI patients. Type III patients showed greater movement of the abdomen during VT, while Type I-IV were more significant on the sides of the rib cage both in VT and IC. No major differences were present during VC.

Figure 4: Colormaps (a, c, f, h, k, m) and arrow plots (b, d, g, i, l, n) of OI Type III patients and OI Type I-IV patients for the three volume variations considered: Tidal Volume (a-d), Inspiratory Capacity (c, d), and Vital Capacity (e, f). Statistical colormaps of the p-value are represented (e, j, o) with red colour indicating points where the OI Type III patients presented a greater average displacement than OI Type I-IV patients, and blue for the opposite situation.

## Discussion

This was the first attempt to analyse vital capacity manoeuvre in OI patients from a global (i.e.: lung and chest wall) to a local level. In this way, it was possible to provide an innovative way to visualize and investigate trunk geometry and kinematics. In OI patients, the kinematics of the trunk changed to compensate for the severe structural deformities by shifting the expansion in the abdomen both at rest and during maximal manoeuvre because of a restricted thorax. This strategy enhanced with the severity of the disease. In addition, paradoxical thoracic movement was present in Type III patients and in was located in the lateral part of the ribcage both during maximal inspiration, but also during full expiration.

OI is known to be a restrictive lung disease [4, 11–15, 29]. This was confirmed by the lower values of FVC and TLC found in all our patients, with Type III OI patients being more restricted compared to Type I-IV patients.

Both volumes were lower in Type III OI when expressed as absolute volumes. This was somewhat expected, since these patients were short individuals, their absolute volumes would be consequently smaller compared to controls and compared to the OI Type I-IV subjects. When expressed as percentage of the predicted values, both volumes of Type III patients were below 70%, therefore revealing the restriction. However, the calculation of predicted normal values for pulmonary function in OI adults still remains an issue, particularly in more severe forms of this condition because of the major dependence on height in such calculation. Individuals with Type III OI and many individuals with Type IV OI tend to be of extreme short stature but their chest cavity might not be proportionately reduced in volume. Arm span cannot be used in place of height in adults with severe OI because they tend to have multiple upper extremity fracture, rodding, and bowing of these extremities. Special attention should be paid on choosing the algorithm for calculating the predicted spirometric values to avoid serious errors in both under and over diagnosis [30]. Artifactual FVC values higher than expected in OI were previously highlighted, leading to potential misinterpretation and overestimation of the real pulmonary function of these patients [15, 31]. We have referred to Global Lung Function Initiative (GLI), that developed reference equations for spirometry [32], carbon monoxide transfer factor [33] and static lung volumes [34], because it is now the largest resource for reference values for routine lung function testing. However, the GLI normal values require no musculoskeletal abnormalities that would affect “true stature”. In absence of specific reference values for patients with skeletal abnormalities, we have chosen the GLI equations because they use flexible statistical methods, they allow non-linear relationship to be described across all ages, they define the normal values of a wide range of ages as well as a more accurate lower limit of normal. For these reasons, the GLI equation are quickly becoming the gold standard for lung function outcomes. The prospective is to shift the interpretation of lung function results from the use of percent predicted to z-scores, as they take into account the predicted value, as well as the between-subject variability of measurements [35]. Dedicated studies are therefore needed to evaluate if z-score normalization might overcome (at least partially) the above-mentioned limitations of the predicted equation in OI. Clinicians should therefore critically interpret excessively good pulmonary function in OI adults, particularly in the most severe form of the disease, as it might be an inaccurate result, resulting from the prediction equation they use.

In addition, FVC and TLC are global volumes that cannot localize the restriction. According to the pathophysiology of OI, the restriction would be expected to be confined in the thorax because of the severe deformity that might affect the spine and the ribcage.

Taking into account all the geometrical quantities that we have measured, it was possible to notice several differences between healthy controls and OI patients. These differences concerned the overall trunk shape and its compartments. The results were similar to our previously presented data on another group of OI patients [15], in terms of both absolute average values and statistical significance. In the case of the OI group, the influence of pathology severity played a role in the most relevant results also of the shape analysis. There was agreement also in the ventilatory pattern data (minute ventilation, breathing frequency, and tidal volume) with previously published paper [15], with the most severe form being characterized by important rapid and shallow breathing at rest and by thoracic restriction. Patients affected by milder forms of OI behaved almost as healthy subjects, even though the magnitude of their breathing pattern was slightly reduced.

Type III patients were characterized by altered thoracic size in terms of reduced height, areas and volumes, but not for the diameters. The ribcage therefore assumed a compressed configuration aggravated by severe scoliosis (both thoracic and lumbar). The main consequence at rest was a systematic thoracic paradoxical breathing in supine position in the most severe form (data non presented because out of the porpoise of our aims) and this might be related to the worst sleep quality of these patients as already found [29]. Posture is known to play a role in the compartmental contribution with vertical position being characterized by increased thoracic breathing. The null contribution of the pulmonary ribcage at rest found in Type III patients in seated position confirmed the thoracic restriction to be present even during daily normal activity. The abdomen motion was prevalent both at rest and during maximal inspiratory and expiratory expansion, presumably because it is not constricted, therefore confirming that the restriction is located in the thorax. It is interest to notice that the thoraco-abdominal strategy (i.e.: the distribution of the movement between the compartments) adopted by healthy subjects and Type I-IV patients remained invariant between resting and maximal breathing.

By contrast, in Type III patients, this occurred between resting breathing and vital capacity, while the thoracic contribution increased during inspiratory capacity and consequently the abdominal (diaphragmatic) contribution reduced, although remaining prevalent.

From the point of view of the respiratory muscles this could be explained by the ribcage muscles to find less difficulties in maximally inflating the ribcage rather than in maximally deflating it; while the action of abdominal muscles seemed to be preserved by the disease. But these are merely speculations and dedicated studies should be focused on directly studying the respiratory muscles in OI.

Although the compartmental analysis of the chest wall brought these new important considerations, some limitations are still present. Considering the respiratory analysis, OEP is able to capture the overall motion of the chest wall with great precision, since it uses a high number of markers that entirely cover the thoraco-abdominal surface. Despite the high accuracy, respiratory analysis with OEP can only focus on the global behaviour of the chest wall compartments at most. What it is missing is the ability to provide information about local kinematics, shifting the point of view from the whole trunk to single points on the surface. New information about the displacement and direction of motion of each point composing the models was extracted from the analysis of the colormaps and the arrow plots. The new proposed approach allowed to investigate chest wall kinematics to a more local level with the models revealing that, in Type III patients, respiratory paradoxes occurred on the side of the rib cage region during resting and maximal inspiratory and expiratory expansions. While globally the thoracic compartment expanded during maximal inspiration and expiration, locally there were areas that moved paradoxically therefore wasting part of the contraction of the respiratory muscles in distorting the ribcage. For the very first time we have localized and quantified the site of the restriction in these patients. These different respiratory strategies found therefore might compensate for the limitations caused by the chest wall deformities.

The thoraco-abdominal compartmental analysis and the average 3D models during vital capacity represented a strength of the study as they allowed for quantify and localize the restriction in these patients. Moreover, the statistical colormaps constituted another strength, being a very intuitive and direct method to enhance the differences among the three groups. More in detail, the approach described presented two peculiar aspects: the precise reconstruction of the thoraco-abdominal surface and the high reproducibility of the process of creation and analysis of the 3D models. The models improved OEP accuracy by increasing the number of points that constituted the surface of the models. In addition, the original OEP points were maintained in the new version of the surface. Since these points corresponded to precise anatomical reference referees, their presence in the new model was another great advantage and allowed to keep the information about global trunk geometry.

Although the focus of this study was on the impact of chest deformities on respiration in OI patients, there is new evidence in the literature that reduced pulmonary function can also be caused by a primary pulmonary problem due to intrinsic collagen alterations. Indeed, type I collagen defects play a crucial role in the development (and defects) of the lung parenchyma [4]. Khan et al., failed to find a relationship between decreased pulmonary function and the severity of scoliosis in OI. They concluded that restrictive lung pattern in this population might not be entirely attributed to thoracic cage deformities [36]. Other factors might play a role, with intrinsic lung disease potentially contributing more than scoliosis [4, 37]. While these new pathological lung mechanisms were only postulated in OI patients, different mouse models of OI have shown the presence of primary lung morphological changes accompanied by functional changes [38–40]. We might speculate that lung function in OI deteriorates further when intrapulmonary defects are combined with severe sternal, thoracic and spinal deformities. These new aspects have crucial clinical and diagnostic implications for the assessment and treatment of the pulmonary issues in OI. In this scenario, the local mapping technique that we are proposing could help to quantify the contributions of chest wall abnormalities, in addition to lung abnormalities, being both contributors to the respiratory issues affecting adults with OI, with imaging used to evaluate alterations and dishomogeneities in lung function imaging. In particular, CT or MR scans obtained at different lung volumes, properly registered [41, 42], can provide regional ventilation maps, so giving information which is related to changes in lung compliance, even at a local level.

## Conclusion

The methods presented improved and extended the characteristics of OEP analysis, providing useful tools for the study of thoraco-abdominal surface geometry and kinematics during respiration. Comparative analyses were also possible, in order to investigate the presence of relevant differences between subjects with various conditions. Our results encouraged the potential extension of this new method to other pathology or conditions characterized by chest wall deformities (i.e.: severe scoliosis, neurodegenerative disease, obesity, demolitive thoracic surgery) with possible important implications.

To conclude, Type III patients are characterized by altered thoracic geometry (in terms of perimeters, areas and volumes), by more severe scoliosis, by rapid and shallow breathing at rest, by more restrictive lung pattern with the restriction localized in the thorax that it is further limited by paradoxical movement of the lateral part of the ribcage. The 3D analysis proposed seemed a promising new method for pathophysiology study of chest wall restriction and/or to study the effect of treatments (surgery, bracing, rehabilitation) aimed to improve chest wall motion.

## Data Availability

The data that support the findings of this study are available on request from the corresponding author ALM. The data are not publicly available due to restrictions, since their containing information that could compromise the privacy of research participants.

## Abbreviations

AHI: apnoea and hypopnea index
AP: Antero-Posterior
FEV_1_: forced expiratory volume in one second
FVC: forced vital capacity
IC: inspiratory capacity
ML: Medio-Lateral
MV: minute ventilation
OEP: Opto-Electronic Plethysmography
ODI: oxygen desaturation index
OI: Osteogenesis Imperfecta
RR: respiratory rate
TLC: total lung capacity
VC: vital capavity
VF: vector field
VT: tidal volume
